# Preventing hemorrhoids during pregnancy: a multicenter, randomized clinical trial

**DOI:** 10.1186/s12884-022-04688-x

**Published:** 2022-04-30

**Authors:** Tomas Poskus, Zivile Sabonyte-Balsaitiene, Lina Jakubauskiene, Matas Jakubauskas, Ieva Stundiene, Gabija Barkauskaite, Mante Smigelskaite, Eugenijus Jasiunas, Diana Ramasauskaite, Kestutis Strupas, Grazina Drasutiene

**Affiliations:** 1 Clinic of Gastroenterology, Nephrourology, and Surgery, Institute of Clinical Medicine, Faculty of Medicine, Vilnius University, Vilnius, Lithuania; 2 Clinic of Obstetrics and Gynecology, Faculty of Medicine, Vilnius University, Vilnius, Lithuania; 3 Faculty of Medicine, Vilnius University, Vilnius, Lithuania; 4 Center of Informatics and Development, Vilnius University Hospital Santaros Klinikos, Vilnius, Lithuania

**Keywords:** Pregnancy, Hemorrhoids, Nutrition, Prevention

## Abstract

**Background:**

To compare specific dietary and behavioral recommendations for hemorrhoids prevention during pregnancy.

**Methods:**

This was a randomized, single-blind, multicenter trial conducted in three different clinical centers. Patients were randomly allocated into two groups in a ratio of 1:1. Intervention consisted of specific dietary and behavioral counseling. The primary outcome of this study was the rate of hemorrhoids at the time of discharge from the obstetrics unit. Categorical variables were compared by the Chi-Squared or Fisher exact tests, as appropriate. Continuous variables were compared using either the Student's t-test or the Mann–Whitney U test. Binary logistic regression model was used to identify independent predictors of hemorrhoids after delivery. This analysis was performed on factors with a *p*-value < 0.10 in univariate analysis. Statistical analysis was performed using IBM SPSS 23.0 and GraphPad Prism 9 software. A *P*-value of less than 0.05 was considered significant for all tests.

**Results:**

We observed a significantly lower hemorrhoids rate in the intervention group at the time of discharge from the obstetrics unit after delivery (intention-to-treat (ITT) (the relative risk (RR) 0.38; 95% the confidence interval (CI) 0.24–0.59; *p* < 0.001) per-protocol (PP) (RR 0.42; 95% CI 0.27–0.64; *p* < 0.001). There was no significant difference in spontaneous miscarriage rate between the groups for both ITT and PP analysis. Additional binary logistic regression analysis revealed that the intervention applied in this study was the only protective factor. Both, the history of hemorrhoids before pregnancy and the increase of newborn height was associated with a higher risk of hemorrhoids.

**Conclusions:**

Our suggested intervention, aimed to modify dietary and behavioral habits, significantly reduces the rate of hemorrhoids after pregnancy and can be safely recommended to pregnant women.

**Trial registration:**

Date of registration: 2016–05-09; Date of initial patient enrollment: 2016–06-02; Trial registration number: 158200–16-843–357; Trial registration site URL: https://www.mf.vu.lt/mokslas/vilniaus-regioninis-biomedicininiu-39tyrimu-etikos-komitetas#isduoti40vrbtek-leidimai.

**Supplementary Information:**

The online version contains supplementary material available at 10.1186/s12884-022-04688-x.

## Background

Hemorrhoids are described as the abnormal downward displacement of the anal cushions causing venous dilatation [1]. The main reported symptoms caused by hemorrhoids are burning, itching, perianal pain and bleeding [2]. This condition is especially prevalent in pregnancy, mainly during the third trimester and the postpartum period [3, 4]. A few clinical studies reported the incidence of hemorrhoids, varying from around 15% to 41%, or even reaching 85% in some of the populations with the tendency to be more common with increased age and parity [3, 5–8].

Several physiological factors are known to provoke hemorrhoids in pregnancy. Increased circulating blood volume and the rise of intraabdominal pressure due to the enlargement of the uterus, cause venous stasis in the perianal region [9, 10]. Moreover, pregnancy hormone progesterone tends to relax smooth muscles not only in the venous walls but also in the intestine, causing reduced motility and further constipation [9]. Some of these factors were acknowledged in several prospective studies. Poskus et al. reported that the personal history of perianal disease, straining during delivery for more than 20 min, birth weight of newborn > 3800 g and constipation are independent risk factors for hemorrhoids and anal fissures [3]. Ferdinande et al. determined that constipation and history of anal problems are significant risk factors for developing perianal disease during pregnancy [5].

Although constipation is one of the best-known modifiable risk factors strongly associated with the development of hemorrhoids during pregnancy, the literature on this topic is scarce. Currently, there are no studies analyzing dietary and behavioral interventions to decrease the rate of hemorrhoids in pregnancy.

The aim of this study was to evaluate the safety and effectiveness of dietary and behavior interventions for pregnant women for the prevention of hemorrhoids during pregnancy and after delivery.

## Materials and methods

### Trial design

A randomized, single-blind, multicenter trial was conducted between June 2016 and June 2019 in three different clinical centers (Vilnius University Hospital Santaros Klinikos; Vilnius City Clinical Hospital and Vilnius Maternity Hospital). Women in early pregnancy (less than 12 weeks of gestation) were informed about the study. If they showed interest in participating, they were screened for eligibility. The study was approved by the Vilnius Regional Bioethics Committee, Vilnius, Lithuania on the 10th of May 2016, registration number 158200–16-843–357 (registration certificate provided as a supplementary document).

### Inclusion and exclusion criteria

Women with early viable pregnancy (less than 12 weeks of gestation) at the age between 18–45 years and who gave written consent were included in this trial. All other women who did not fulfill all the inclusion criteria were not eligible to participate in the study.

### Randomization

Patients were randomly allocated into two groups in a ratio of 1:1. A computer-based randomization sequence was generated, transferred and sealed into individual envelopes. Once a patient gave written consent to participate a clinician unsealed an envelope in sequence and the patient was allocated into one of the two groups.

### Intervention

The study intervention was designed by using the following guidelines and recommendations [11, 12]. During the first visit, each woman in the intervention group received a structured 30-min personal consultation with written instructions of dietary and behavioral recommendations. Women were advised to eat at regular time intervals; Consume at least 1.5 L of fluid, avoid food, that causes constipation; Consume a tablespoon of bran and 2–5 prunes daily; Consume around 300 g of fruits, 500 g of vegetables and 30 g of nuts daily; Exercise and/or walk daily 30–60 min, 3–5 times per week. Furthermore, there were specific recommendations for defecation: not to ignore the urge to defecate; Spend less than 3 min on the commode; Attempt to defecate 30–40 min after eating and in the mornings; Washing after bowel movement. Full detailed intervention recommendations are provided as supplementary material. Women in the control group received standardized, nationally approved physical activity and dietary recommendations for pregnant women.

### Study visits and data collection

**Fig. 1:**
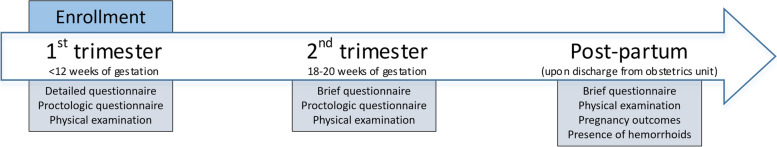
Timeline of the study visits

Each participant had a total of three study visits (Fig. 1.). The first visit took place during the first trimester of pregnancy (< 12 weeks of gestation), second visit during the second trimester of pregnancy (18–20 weeks of gestation) and the third visit was carried out upon discharge from the obstetrics unit 2–3 days after childbirth. The First visit coincided with the study enrollment, during which a detailed questionnaire consisting of socioeconomic factors, physical activity, anthropometric data (weight and height), obstetric history, perianal symptoms in previous pregnancies and the presence of chronic health conditions was filled out. The proctologic questionnaire was filled out and the physical examination was performed during each visit. On the first and second visits, detailed information about dietary habits, physical activity, alcohol and tobacco consumption was collected. Pregnancy outcomes and neonatal data were gathered from medical records during the third visit.

### Outcomes and blinding

The primary outcome of this study was the rate of hemorrhoids at the time of discharge from the obstetrics unit. Outcome was assessed by a gynecologist who remained blinded to the patients’ allocation group. To keep the evaluation of the outcome measure consistent, all participating gynecologists underwent a 4-h seminar conducted by the same expert proctologist on how to assess the presence of hemorrhoids using a standardized methodology.

Secondary outcomes of the study were the safety of the intervention, measured by the rate of miscarriages in both groups, and the possible risk factors to develop hemorrhoids during pregnancy.

### Sample size calculation

The sample size was calculated using the G*Power software. We presumed the baseline risk of hemorrhoids during pregnancy of 35% from our previous experience and we predicted that the intervention would reduce the risk to 17% [3]. Based on the statistical power of 80% and a level of significance set at 5% we calculated the total sample size to be 206 patients. The sample size was increased by 30% to 260 patients to account for loss to follow-up and miscarriages. As the intervention: control ratio was 1:1, each arm of the study consisted of 130 participants.

### Statistical analysis

Categorical variables were compared by the Chi-Squared or Fisher exact tests, as appropriate. Continuous variables were compared using either the Student's t-test or the Mann–Whitney U test. Binary logistic regression model was used to identify independent predictors of hemorrhoids after delivery. This analysis was performed on factors with a *p*-value < 0.10 in univariate analysis. Statistical analysis was performed using IBM SPSS 23.0 and GraphPad Prism 9 software. A *P*-value of less than 0.05 was considered significant for all tests.

## Results

**Fig. 2:**
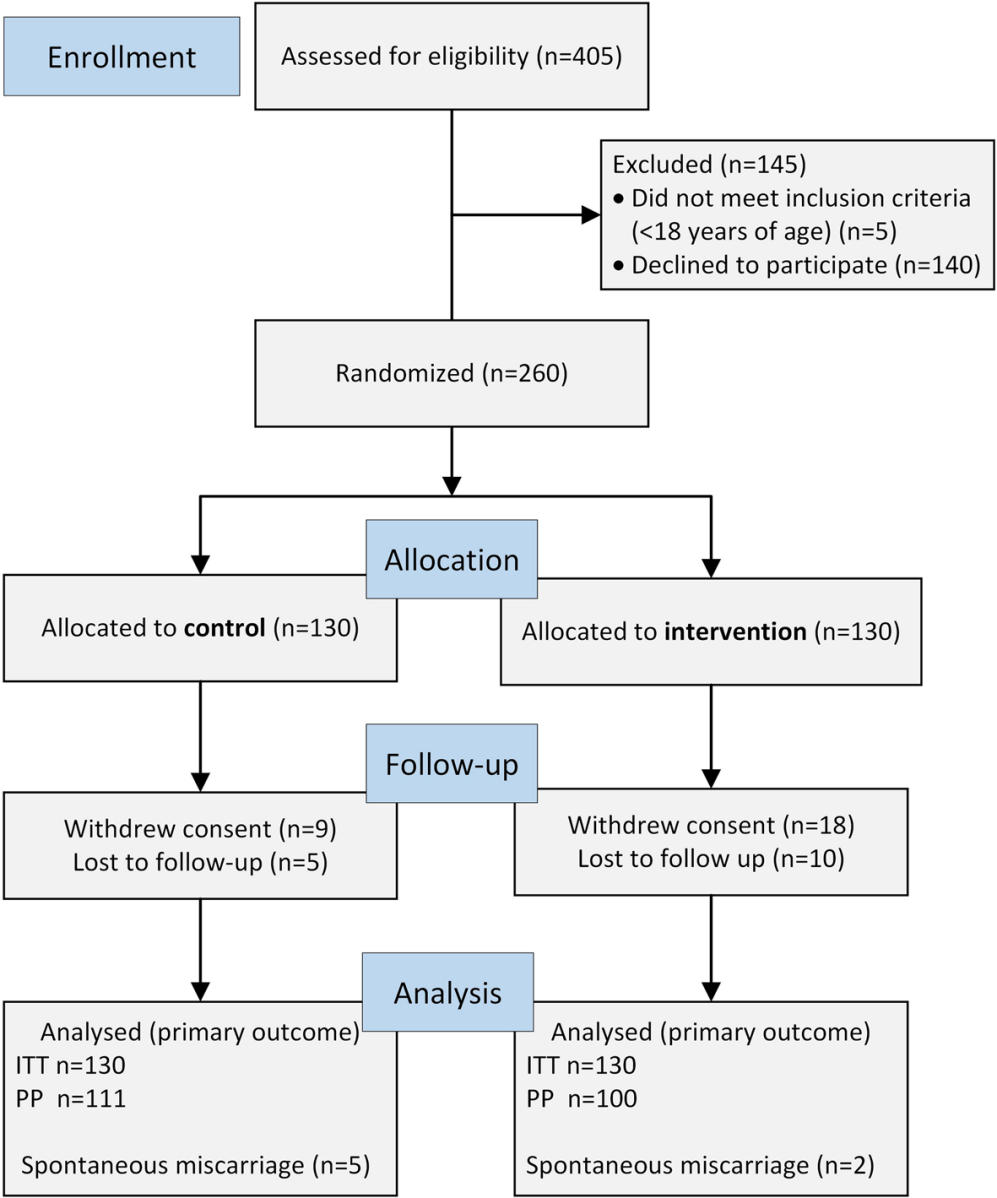
CONSORT flowchart of the study

Between June 1st, 2016 and June 1st, 2019, a total of 405 pregnant women were screened for eligibility. Of these, 260 were randomly assigned to either intervention (*n* = 130) or control (*n* = 130) group (Fig. 2.). There were 14 (10.8%) women in the control and 28 (21.5%) in the intervention group, who did not finish the study.

Baseline demographic, obstetric and coloproctological characteristics of all randomized women are presented in Table 1. There were a few minor differences between the groups. Women in the intervention group had a higher education compared to controls. Moreover, perianal pain was more prevalent between women in the intervention group than in the control group (6 (4.6%) vs. 16 (12.3%), *p* = 0.026). Otherwise, there were no additional statistically significant differences between the groups at the start of the study.

**Table 1:** Baseline characteristics by randomized group

	**Control (*N* = 130)**	**Intervention (*N* = 130)**	***P* value**
**Demographic variables**			
Age [mean ± SD]	30.3 ± 4.6	30.1 ± 4.5	0.746
BMI (before pregnancy) [median [Q1;Q3]]	22.1 [20.7;25.0]	21.5 [19.8;24.4]	0.083
Marital status [n (%)]			0.140
Married	95 (73.1)	102 (78.5)	
Partnership	26 (20.0)	15 (11.5)	
Lonely	9 (6.9)	13 (10.0)	
Education [n (%)]			0.012
Secondary	16 (12.3)	17 (13.1)	
Special secondary	22 (16.9)	6 (4.6)	
Unfinished higher	14 (10.8)	21 (16.2)	
Higher	78 (60.0)	86 (66.2)	
Living conditions [n (%)]			0.516
Satisfactory	21 (16.2)	25 (19.2)	
Good	109 (83.8)	105 (80.8)	
Living area [n (%)]			0.201
Rural	28 (21.5)	20 (15.4)	
Urban	102 (78.5)	110 (84.6)	
Monthly income [n (%)]			0.653
< 300 euro	10 (7.7)	12 (9.2)	
300–500 euro	34 (26.2)	28 (21.5)	
> 500 euro	86 (66.2)	90 (69.2)	
Physical activity [n (%)]			0.901
Too low	70 (53.8)	68 (52.3)	
Enough	60 (46.2)	62 (47.7)	
**Obstetric variables**			
Menarche [median [Q1;Q3]]	13.0 [12.0;14.0]	13.0 [12.0;14.0]	0.855
Number of previous pregnancies [n (%)]			0.141
0	63 (48.5)	53 (40.8)	
1	44 (33.8)	38 (29.2)	
2	17 (13.1)	28 (21.5)	
3 and more	6 (4.6)	11 (8.5)	
Outcomes of previous delivery [n (%)]			0.536
Did not give birth	72 (55.4)	63 (48.5)	
Vaginal delivery	46 (35.4)	53 (40.8)	
Cesarean delivery	12 (9.2)	14 (10.8)	
Previous perineal tear [n (%)]	16 (12.3)	14 (10.8)	0.698
Previous episiotomy [n (%)]	26 (20.0)	26 (20.0)	1.000
**Coloproctological variables**			
History of hemorrhoids [n (%)]	13 (10.0)	15 (11.5)	0.842
Current perianal discomfort [n (%)]	29 (22.3)	33 (25.4)	0.560
Current perianal pain [n (%)]	6 (4.6)	16 (12.3)	0.026
Current perianal bleeding [n (%)]	5 (3.8)	9 (6.9)	0.272
Current perianal lumps [n (%)]	14 (10.8)	14 (10.8)	1.000
Constipation [n (%)]	20 (15.4)	30 (23.1)	0.116
History of perianal operations [n (%)]	1 (0.8)	3 (2.3)	0.622
Family history of perianal disease [n (%)]	25 (19.2)	32 (24.6)	0.294

Pregnancy outcomes of women who fully completed the study are reported in Table 2. Both study groups were equal according to the delivery and newborn parameters.

**Table 2:** Pregnancy outcomes

	**Control (*N* = 111)**	**Intervention (*N* = 100)**	***P* value**
Weight gain (kg) [median [Q1;Q3]]	13.0 [10.0;16.0]	14.0 [11.0;17.0]	0.113
Gestational diabetes [n (%)]	13 (11.7)	7 (7.0)	0.347
Birth week [median [Q1;Q3]]	39.0 [39.0;40.0]	39.0 [38.0;40.0]	0.801
Preterm birth [n (%)]	12 (10.8)	12 (12.0)	0.831
Birth assistance [n (%)]			0.182
Vaginal birth without assistance	87 (78.4))	67 (67.0)	
Vaginal birth with assistance	4 (3.6)	6 (6.0)	
Cesarean delivery	20 (18.0)	27 (27.0)	
Newborn weight (g) [median [Q1;Q3]]	3520.0 [3200.0;3840.0]	3570.0 [3100.0;3925.0]	0.840
Newborn height (cm) [median [Q1;Q3]]	52.0 [51.0;58.0]	53.0 [51.0;55.0]	0.339
Head circumference (cm) [median [Q1;Q3]]	35.0 [34.0;36.0]	35.0 [34.0;36.0]	0.466

The primary outcome was analyzed for both intention-to-treat (ITT) and per-protocol (PP) populations and is presented in Table 3. We observed a significantly lower hemorrhoids rate in the intervention group at the time of discharge from the obstetrics unit after delivery (ITT (risk ratio (RR) 0.38; 95% confidence interval (CI) 0.24–0.59; *p* < 0.001) PP (RR 0.42; 95% CI 0.27–0.64; *p* < 0.001).

**Table 3:** Rate of hemorrhoids at the final visit

	**Control group**	**Intervention group**	**Relative risk [95% CI]**	***P* value**
Hemorrhoids rate (ITT)	53/130 (40.8%)	20/130 (15.4%)	2.65 [1.71–4.19]	** < 0.001**
Hemorrhoids rate (PP)	53/116 (47.7%)	20/102 (20.0%)	2.39 [1.56–3.73]	** < 0.001**

Five cases of spontaneous miscarriages in the control group and two cases in the intervention group were observed during the study period. Rates of spontaneous miscarriages are presented in Table 4. There was no significant difference between the groups for both ITT and PP analysis.

**Table 4:** Rate of spontaneous miscarriages by randomized group

	**Control group**	**Intervention group**	**Relative risk [95% CI]**	***P* value**
Spontaneous miscarriage rate (ITT)	5/130 (3.8%)	2/130 (2.7%)	0.40 [0.09–1.75]	0.447
Spontaneous miscarriage rate (PP)	5/116 (4.3%)	2/102 (2.0%)	0.45 [0.10–1.98]	0.452

Binary logistic regression analysis revealed independent risk factors for developing hemorrhoids after delivery (Table 5). Intervention applied in this study was the only protective factor (OR 0.171, 95%CI 0.081 – 0.361, *p* < 0.001). History of hemorrhoids before pregnancy greatly increases the chance to develop hemorrhoids after giving birth (OR 15.192, 95%CI 1.843–125.228, *p* = 0.011). Moreover, the increase of newborn height was associated with a higher risk of hemorrhoids (OR 1.282, 95%CI 1.026–1.603, *p* = 0.029).

**Table 5:** Multivariate logistic analysis of risk factors for developing hemorrhoids

Risk factor	OR	95% CI	*P* value
Intervention	0.171	0.081 – 0.361	** < 0.001**
History of haemorrhoids	15.192	1.843–125.228	**0.011**
Baseline perianal discomfort	0.870	0.265 – 2.854	0.819
Baseline perianal pain	1.378	0.202 – 9.386	0.743
Baseline perianal lumps	1.916	0.206 – 17.836	0.568
Baseline perianal bleeding	2.536	0.346 – 18.618	0.360
Newborn height	1.282	1.026 – 1.603	**0.029**
Newborn weight	0.999	0.998 – 1.001	0.452
Newborn head circumference	0.907	0.663 – 1.241	0.542

## Discussion

### Principal findings

Our study is the first randomized controlled trial to prove that a counselling intervention, aimed to modify dietary and behavioral habits, can significantly reduce the rate of hemorrhoids in pregnancy. Both ITT and PP analyses showed that this intervention managed to decrease the hemorrhoids rate by about half (ITT (RR 0.38; 95% CI 0.24–0.59; *p* < 0.001) PP (RR 0.42; 95% CI 0.27–0.64; *p* < 0.001). The incidence of hemorrhoids after giving birth in the control group (ITT-40.8%; PP-47.7%) was in line with the reported rate of 40.7% observed by Poskus et al. in a similar population [3].

### Clinical and research implications

Pregnant women are a very vulnerable population therefore the safety of intervention is crucial. We chose to analyze the miscarriage rate in order to prove that our intervention did not prompt unfavorable pregnancy outcomes. The miscarriage rate in both groups did not differ significantly, furthermore, the patients did not report any additional side-effects that could be attributed to the effects of an intervention.

We identified that the history of perianal disease and newborn height were independent risk factors to develop hemorrhoids after delivery on multivariate analysis. Intervention was the only protective factor significantly reducing the likelihood of hemorrhoids. Our findings are somewhat similar to those reported by Ferdinande et al. and Poskus et al. as they also determined that a previous history of perianal disease is highly associated with the increase of hemorrhoids rate during pregnancy [3, 5]. However, we did not find that constipation before the first trimester would be associated with hemorrhoids after delivery.

Intervention applied in our study contained dietary and behavioral habit changes which are also recommended for conservative hemorrhoids treatment in non-pregnant patients [13, 14]. Our intervention is safe, cost-effective and does not require any additional training for the medical personnel or patient. This allows to apply this intervention widely without limiting it to specialised treatment centers. In our opinion, counselling could be a task for primary care as modifying risk factors and changing behaviour when planning a pregnancy may yield even better results.

### Limitations of the study

The main limitation of this study is the number of patients who were lost to follow-up (14 (10.8%) women in the control and 28 (21.5%) in the intervention group). This, perhaps, reflected the population (working age) and the condition (considered by some to be a sensitive subject). Having this in mind, we performed both ITT and PP analyses, which gave us identical results, showing that these lost to follow-up patients did not skew our findings. Furthermore, the study’s initial sample size was increased by 30% to account for loss to follow-up and miscarriages thus the study still had sufficient statistical precision to detect differences between groups. Small differences between study groups may have been missed due to the low miscarriage rates, however, miscarriage rate is the most acknowledged outcome when evaluating the safety of various interventions during pregnancy. Our study design was pragmatic and we did not strictly control whether the patient complied with the intervention’s recommendations. However, as both trial arms were balanced on the baseline characteristic and were equal on pregnancy outcomes, we would draw a conclusion that the intervention had the main influence on the reduced rate of hemorrhoids after delivery.

## Conclusion

In conclusion, our suggested intervention, aimed to modify dietary and behavioural habits, significantly reduces the rate of hemorrhoids in pregnancy and can be safely recommended to pregnant women.

## Supplementary Information

**Additional file 1:** 

## Data Availability

Data availability statement Data are available on reasonable request. All data relevant to the study are included in the article or uploaded as online supplementary information. Deidentified data, that underlie the results reported in this article, will be shared with third parties after written request to the corresponding author describing intention of data usage and full affiliation of the requesting organization. To gain access to the data, a data access agreement need to be signed.

## Abbreviations

RR: The relative risk
CI: The confidence interval
ITT: Intention-to-treat
PP: Per-protocol
BMI: Body mass index
OR: The odds ratio
