# Supplementary material for: Preventing hemorrhoids during pregnancy: a multicenter, randomized clinical trial
Source: BMC Pregnancy Childbirth. 2022 Apr 30;22:374. doi: 10.1186/s12884-022-04688-x (PMC9055760; doi:10.1186/s12884-022-04688-x)
Supplement: Supplementary file 1 — Additional file 1: [file 12884_2022_4688_MOESM1_ESM.docx]

**Dietary and behavioral recommendations for constipation prevention**

1. Plan the time of your meals, try to eat at regular time intervals every 3-4 hours.
2. Consume at least 1.5 liters of water every day. Avoid sparkling water
3. Don’t ignore the urge to defecate
4. Attempt to defecate 30-40 minutes after a meal and in the mornings
5. During defecation place the full sole of your foot on the ground or on a foot stool
6. Exercise and/or walk daily 30-60 minutes, 3-5 times a week
7. Avoid food that causes constipation:
   1. Salty food
   2. Large amount of chocolate, sweets
   3. Fried food
   4. Products from refined flour
   5. Large amounts (> 5 cups per day) of coffee, black/green tea, caffeine containing drinks (i.e. energy drinks)
8. Consume a tablespoon of bran daily. Bran needs to be soaked in hot water for at least 30 minutes before eating.
9. Consume 3-5 soaked prunes daily
10. Consume 20-35 g of fiber rich food daily
11. Consume around 300g of fruits and 500g of vegetables daily
12. Consume around 30g of nuts daily

Choose products containing whole grain:

- Choose whole grain bread instead of bread from refined flour
- Choose brown or red rice instead of white rice
- Choose whole grain pasta instead of pasta from refined flour
- Chose whole grain, spelt or buckwheat flour instead of refined flour

Recommended vegetables:

- Legume vegetables
- Broccoli
- Carrots
- Asparagus
- Root vegetables
- Sweet potatoes
- Onions, Garlic
- Leeks
- Chicory
- Jerusalem artichokes
